# Electrochemical Detection of Penicillin G Using a Nanomolecularly Imprinted Polymer Sensor Modified with Carbon Nanofibers and Gold Nanowires

**DOI:** 10.1021/acsomega.6c06165

**Published:** 2026-09-24

**Authors:** Suhainie Ismail, Nor Azah Yusof, Zulaiha Abdul Rahim, Siti Fatimah Abd Rahman, Shahrul Ainliah Alang Ahmad, Norhazlin Zainuddin, Muhammad Hafiznur Yunus, Ahmad Farabi Mohamad Saman

**Affiliations:** † Institute of Nanoscience and Nanotechnology, 37449 Universiti Putra Malaysia, 43400 Serdang, Selangor, Malaysia; ‡ Chemistry Department, Faculty of Science, Universiti Putra Malaysia, 43400 Serdang, Selangor, Malaysia; § School of Electrical and Electronic Engineering, Tuanku Syed Sirajuddin Engineering Campus, 65271 Universiti Sains Malaysia, 14300 Nibong Tebal, Pulau Pinang, Malaysia; ∥ Institute for Research in Molecular Medicine, Universiti Sains Malaysia, 16150 Kubang Kerian, Kelantan, Malaysia

## Abstract

This study explores the electrochemical behavior of nanomolecularly imprinted polymers (NMIPs) integrated with carbon nanofibers (CNFs) and gold nanowires (AuNWs) modified on screen-printed carbon electrodes (SPCEs) using cyclic voltammetry, electrochemical impedance spectroscopy, and differential pulse voltammetry for the determination of penicillin G. The incorporation of CNFs and AuNWs enhanced electron transfer and improved the electrocatalytic performance of the modified sensor. NMIPs act as artificial biomarkers that selectively bind to the target analyte, penicillin G. Bare and modified SPCEs were characterized using [Fe­(CN)_6_]^3–^/^4–^ as a redox probe in 1 M KCl. The chemical composition and surface morphology of the nanomaterials were further characterized using field-emission scanning electron microscopy, transmission electron microscopy, Fourier transform infrared spectroscopy, and X-ray diffraction analysis. The CNFs/AuNWs/SPCE-modified sensor showed a 2.35-fold increase in current compared with the bare electrode. The fabricated CNFs/AuNWs/NMIPs/SPCE sensor demonstrated a strong linear response over the range of 0.001–30 μM (*R* ^2^ = 0.9892), with a detection limit of 1.0 nM. Furthermore, the sensor exhibited excellent selectivity toward penicillin G, along with good reproducibility (1.14%), stability, and satisfactory recovery rates (92%–98%) in pharmaceutical products. This sensor shows significant potential for penicillin G detection and may be applied in future pharmaceutical industry applications.

## Graphical abstract

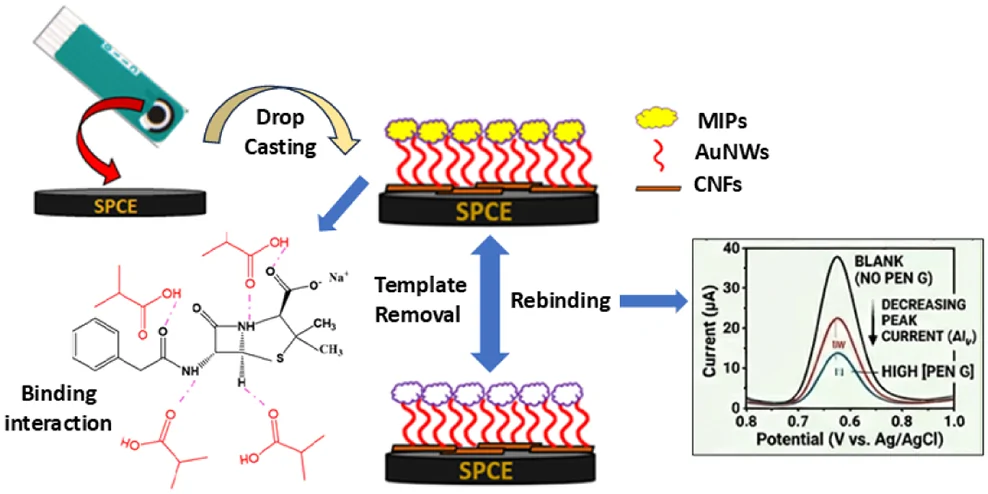

## 1 Introduction

Drug allergy has become a global public health concern. According to statistics, up to 1,000 deaths per year worldwide are associated with drug allergies.^1^ Penicillin allergy is one of the most common causes of adverse drug reactions, such as anaphylaxis.^2^ Penicillin G is an effective semisynthetic broad-spectrum β-lactam antibiotic widely used to treat numerous bacterial infections.^3^ Despite its effectiveness against bacteria, Penicillin G is also a well-known allergen that can trigger serious adverse reactions in humans, including anaphylaxis.^4^ Additionally, exposure to this sensitizing agent may cause life-threatening symptoms such as respiratory problems, skin rashes, and eye irritation.^5^

Cross-contamination of nonpenicillin drugs with penicillin during pharmaceutical manufacturing can also contribute to drug allergies. Such contamination may cause unintended exposure and severe allergic reactions, such as hypersensitivity.^6^ Accordingly, all nonpenicillin products must undergo contamination testing before release, and contaminated drug products should not be marketed or distributed to ensure patient safety. Therefore, reliable, low-cost, rapid, and simple analytical methods are urgently needed to monitor penicillin contamination in nonpenicillin pharmaceutical products before distribution to consumers.

Conventional analytical approaches, including high-performance liquid chromatography, liquid chromatography–mass spectrometry, fluorescence spectroscopy, and surface-enhanced Raman spectroscopy, present major drawbacks and limitations. These methods are time-consuming, expensive, and require complicated instrumentation.^7^ By contrast, electrochemical sensors have become a cornerstone of modern analytical chemistry, offering a versatile and efficient approach to detecting and quantifying chemical species by measuring electrical signals.^8^ As demand for efficient, reliable analytical tools continues to grow, electroanalytical techniques remain at the forefront of innovation, driving advances across numerous scientific and technological fields. These techniques are widely used in environmental assessment, clinical diagnostics, food safety monitoring, and industrial process control because of their simplicity, cost-effectiveness, and ability to enable real-time analysis.^9^ Numerous electrochemical approaches have been reported for determining penicillin. For instance, Salihu et al. developed a nickel nanoparticle-modified electrode specifically for the electrochemical detection of Penicillin G in bovine milk. Incorporating the nickel nanocomposite significantly enhanced the electrochemical response to Penicillin G, enabling sensitive detection even at trace concentrations.^10^ Hoa et al. presented a novel approach to green electrochemical sensing using reduced graphene oxide (rGO)-modified electrodes to detect ampicillin (AMP).^11^ The reduced graphene oxide/glassy carbon electrode (rGO/GCE) sensor provides a sensitive, reliable, and cost-effective solution for detecting AMP in pharmaceutical applications. Moreover, additional research has investigated electrochemical sensors utilizing magnetic mesoporous carbon substrates for the swift identification of penicillin sodium in milk.^12^ The sensor indicates promising potential for food safety detection applications.

Molecularly imprinted polymers (MIPs) are synthetic polymeric materials that mimic biological recognition systems and function based on Fischer’s lock-and-key principle.^13^ Recently, MIPs have received growing attention owing to their ability to act as selective recognition units for numerous target analytes, including biomarkers, proteins, and drugs.^14^ These artificial antibodies can be integrated into electrochemical sensors, providing an effective strategy for the selective detection of target analytes. Thus, MIP incorporation into electrochemical sensors has demonstrated performance in antibiotic detection, specifically for Penicillin G sodium. Recently, MIP-based electrochemical sensors have been reported for various applications in determining different antibiotics. Abera et al. demonstrated the use MIP-modified laser-induced graphene electrodes to detect tetracycline in dairy and meat products.^15^ Yahyapour developed a MIP sensor using a polymer and cometal organic framework nanofiber, in conjunction with a GCE, to determine ciprofloxacin.^16^ Zhang et al. introduced a MIP-based electrochemical sensor that utilizes a PEDOT–OH@Au/MWCNT–OH composite for the ultrasensitive detection of pefloxacin.^17^ This advanced MIP-based sensor indicates remarkable selectivity and sensitivity, making it a powerful tool for monitoring antibiotics.

Despite significant advances in electrochemical sensing of Penicillin G, achieving both high sensitivity and selectivity remains challenging, particularly in distinguishing Penicillin G from structurally related β-lactam antibiotics. Nanomaterial-based electrodes can enhance electrochemical responses but generally lack specific molecular recognition. In contrast, MIP-based sensors provide selective recognition but may suffer from limited conductivity and electron-transfer efficiency. Therefore, integrating Penicillin G-selective MIPs with conductive nanomaterials such as carbon nanofibers (CNFs) and gold nanowires (AuNWs) offers a promising strategy to overcome these limitations.

In recent years, nanomaterials have been widely used in nanotechnology and have emerged as key components in advancing electrochemical sensors. Their unique physical, chemical, and electrical properties at the nanoscale have revolutionized the detection of a wide range of analytes.^18^ In addition, nanomaterials enhance sensor performance by providing a high surface area and excellent electrical conductivity. These characteristics make them ideal candidates for electrode modification, thereby improving the sensitivity, selectivity, and overall efficiency of electrochemical sensors. CNFs are a class of nanomaterials that have attracted significant attention in research owing to their exceptional properties, including high surface area, excellent mechanical strength, and superior electrical conductivity.^19^ Moreover, AuNWs have emerged as a highly promising class of nanomaterials in nanotechnology owing to their unique structural, electronic, and optical properties. These properties, combined with gold’s inherent stability and biocompatibility, make AuNWs highly attractive for fundamental research and practical applications. When integrated into an MIP-based electrochemical sensor, CNF/AuNW modifiers can significantly improve sensitivity and selectivity.

Herein, we developed a CNF/AuNW/MIPs/SPCE to combine selective molecular recognition with enhanced electrochemical performance for sensitive PenG detection in pharmaceutical formulations. This research builds upon prior investigations into electrochemical sensors for Penicillin G and other penicillins, presenting a novel integration of molecular recognition with enhanced electrochemical transduction within a single sensing platform. While earlier sensors predominantly utilized nanomaterials or enzymes to enhance sensitivity, MIP-based sensors have primarily focused on providing selective molecular recognition. The sensor proposed in this study uniquely combines Penicillin G-selective nanomolecularly imprinted polymers (NMIPs) with CNFs and AuNWs on a screen-printed carbon electrode (SPCE). The NMIPs selectively recognize PenG, while the CNFs/AuNWs nanostructure significantly enhances electroactive surface area and electron-transfer capabilities. This integrated architecture thus presents a synergistic combination of selectivity and electrochemical sensitivity, with promising applications for the rapid screening of PenG contamination in pharmaceutical products.

## 2 Materials and Methods

### 2.1 Reagents and Instruments

All aqueous solutions were prepared using 18.2 MΩ·cm ultrapure water from Barnstead Nanopure (Thermo Fisher Scientific, USA), unless stated otherwise. The tetrachloroauric acid (HAuCl_4_), trisodium citrate, 3-aminopropyltriethoxysilane (APTES), and 3,3′-dithiodipropionic acid (DTDPA) used in this experiment are of analytical grade and were purchased from Sigma-Aldrich, St. Louis, USA. Carbon nanofibers (100 nm in diameter, 10 μm length, 99.5% purity) used in this work were purchased from Research Nanomaterials Inc., Houston, TX, USA. All cyclic voltammetry measurements were performed in 5 mM [Fe­(CN)_6_]^3–^/^4–^ with 100 mM KCl.

### 2.2 Apparatus

The electrochemical measurements were conducted using a μAUTOLAB (III) potentiostat (Eco Chemie, Utrecht, The Netherlands) with a screen-printed carbon electrode (SPCE), comprising a carbon counter electrode, an Ag/AgCl reference electrode, and a carbon working electrode. All measurements and analyses were controlled using NOVA 2.1.7 software (2020, Metrohm Autolab BV, Utrecht, The Netherlands). The characterization of the CNFs/AuNWs/MIPs/SPCE surface was carried out using field emission scanning electron microscopy (FESEM) (NOVA NANOSEM 230, FEI, Diepoldsau, Switzerland). The elemental composition of the samples was determined by energy dispersive X-ray (EDX) analysis (Hitachi S-3400N, Tokyo, Japan). The synthesized AuNWs were characterized using high-resolution transmission electron microscopy (HRTEM) (JEOL JEM-2010HR, Tokyo, Japan) and ultraviolet–visible (UV–Vis) spectroscopy (Lambda 35, PerkinElmer, USA).

### 2.3 Preparation of Nanomaterials

CNFs (100 mg) were dispersed in a 1:1 mixture of HNO_3_ (5 M) and H_2_O_2_ (30%) in a two-neck round-bottom flask, equipped with a reflux condenser. The mixture was then sonicated and refluxed at 70 °C for 8 h. After cooling to 25 °C, the paste was diluted with deionized water and filtered through a membrane filter. The collected solids were thoroughly washed with deionized water and filtered again until the filtrate turned colorless. The resulting functionalized CNFs were then dried overnight in a vacuum oven.

For the synthesis of Au nanowires, 4.5 mL of a 0.50 M α-naphthol ethanol solution was mixed with 4.5 mL of a 0.05 M HAuCl_4_ aqueous solution at 60 °C under a water bath. The solution color immediately changed from yellow to black, indicating the rapid reduction of the Au precursor. After aging for about 1 min, the resulting product was collected by centrifugation and washed several times with ethanol. The obtained Au nanowires were then dispersed in ethanol for further characterization.^20^

### 2.4 Preparation of Nanomolecularly Imprinted Polymer

Nanomolecularly imprinted polymer (NMIPs) was prepared using the previous method.^21^ Briefly, AIBN (2,2’-azobis­(isobutyronitrile)) and PenG were added to a mixture of 4 mmol of MAA (monomer methacryclic) and 10 mmol of EGDMA (ethylene glycol dimethacrylate). Next, 323 μL of HD (hexadecane) was added to the mixtures and was stirred for 15 min. In another beaker, 50 mg of SDS (sodium dodecyl sulfate) was dissolved in 50 mL of deionized water, known as the water phase. The mixture was stirred for 15 min. Under high-speed stirring, the oil phase was added to the water phase and was stirred for about 1 h. Next, the mixture was sonicated in an ice–water bath using a probe sonicator. Consequently, nitrogen gas was allowed to flow within the mixture for 15 min to remove excess oxygen. Next, the polymerization was conducted at 70 °C for 16 h. After that, the polymer was freeze-dried for 24 h. The resulting polymers were then washed with water to remove any residual surfactant, costabilizer, and template. Finally, the polymers were vacuum-dried at 50 °C.

### 2.5 Fabrication of CNFs/AuNWs/MIPs/SPCE

The preparation of the molecularly imprinted polymer-based electrochemical sensor is illustrated in Figure 1. Initially, the SPCE was washed with deionized water and dried at room temperature. Next, 5 μL of 3 mg/mL of CNFs was drop-cast onto the surface of the modified electrode and left to dry overnight at room temperature (referred to as CNFs/SPCE). The following day, 3 μL of a 5 mM ethanolic solution of DTDPA was drop-cast onto the modified CNFs/SPCE and incubated at room temperature for 1 h. Afterward, 3 μL of a 5 mg/mL AuNW suspension was drop-cast onto the modified CNFs/SPCE and allowed to dry for 1 h. Next, 3 μL of 2 mM aminothiol was drop-cast onto the modified AuNWs/CNFs/SPCE. Finally, 5 μL of MIPs was drop-cast on the modified electrode. In general, Au exhibits a strong affinity for sulfur atoms. Additionally, molecules with thiol ligands form dative bonds when interacting with metal surfaces and metal ions. Hence, both DTDPA and aminothiol act as bidentate thiol ligands and were employed in this reaction with AuNWs due to their enhanced bonding to the modified electrode surface.^22,23^ As illustrated in Figure 1, f-CNFs refer to CNFs, and NMIPs refer to MIPs.

**Figure 1:**
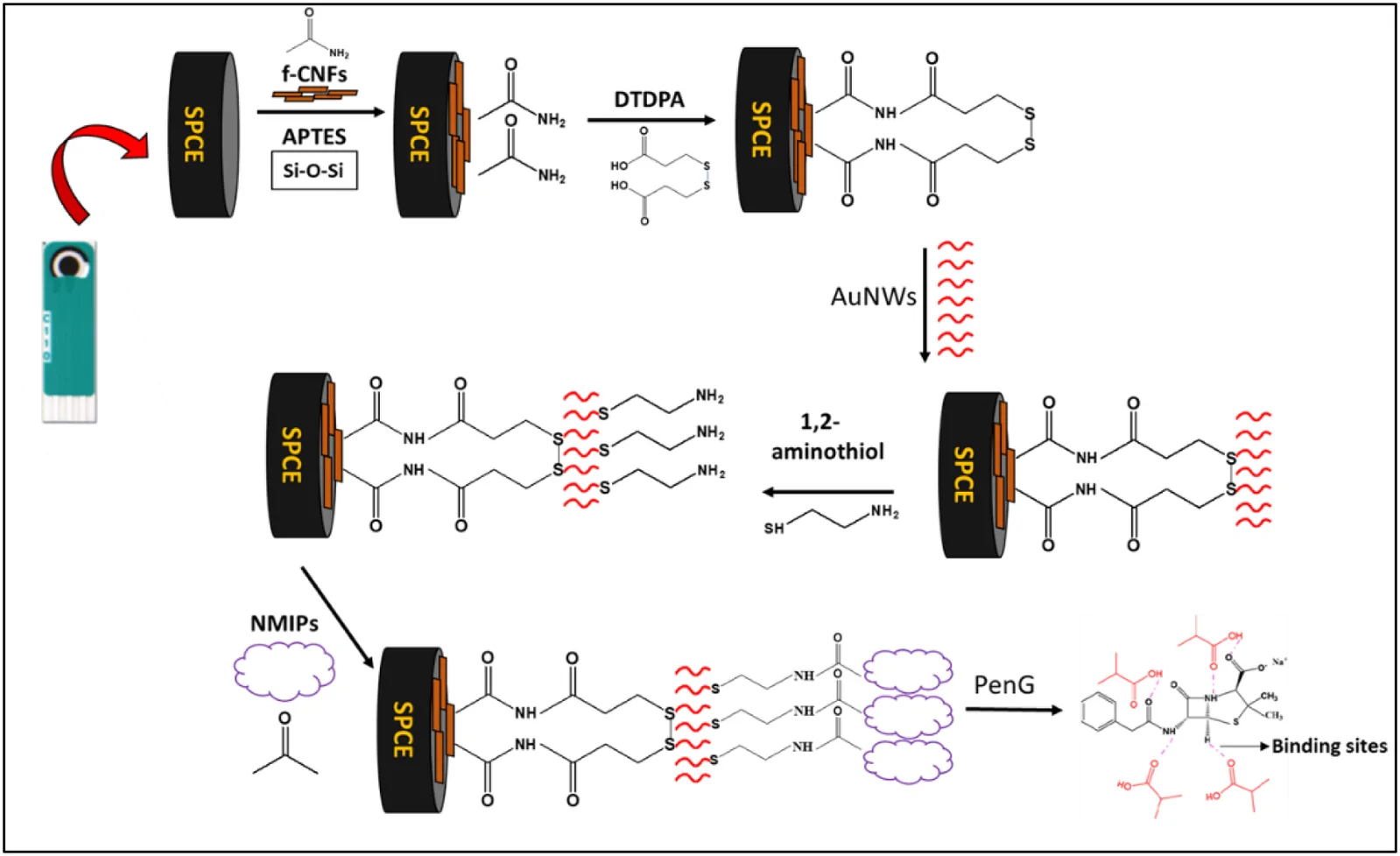
Scheme of the electrochemical sensor fabrication for PenG detection.

## 3 Results and Discussion

### 3.1 Surface Characterization Using HRTEM and FESEM

The HRTEM images of functionalized CNFs are demonstrated in Figure 2A at a magnification of 100 k. The CNFs exhibited the intrinsic fiber-like structure, with an average diameter of 80 nm. This nanoscale enhances the conductivity of carbon nanomaterial, resulting in excellent electrochemical performance.^24^ In addition, the surface morphology of the synthesized functionalized CNFs was analyzed using FESEM, indicating the fibrous structure as highlighted in Figure 2D. Next, the HRTEM image in Figure 2B demonstrates that the gold nanowires (AuNWs) are ultrathin and structurally uniform.^25^ According to the FESEM analysis in Figure 2E, the AuNWs were successfully distributed on the surface of the substrate. Furthermore, HRTEM and FESEM images of MIPs in Figure 2C and F reveal that the polymers are spherical, with diameters of approximately 40–100 nm.

**Figure 2:**
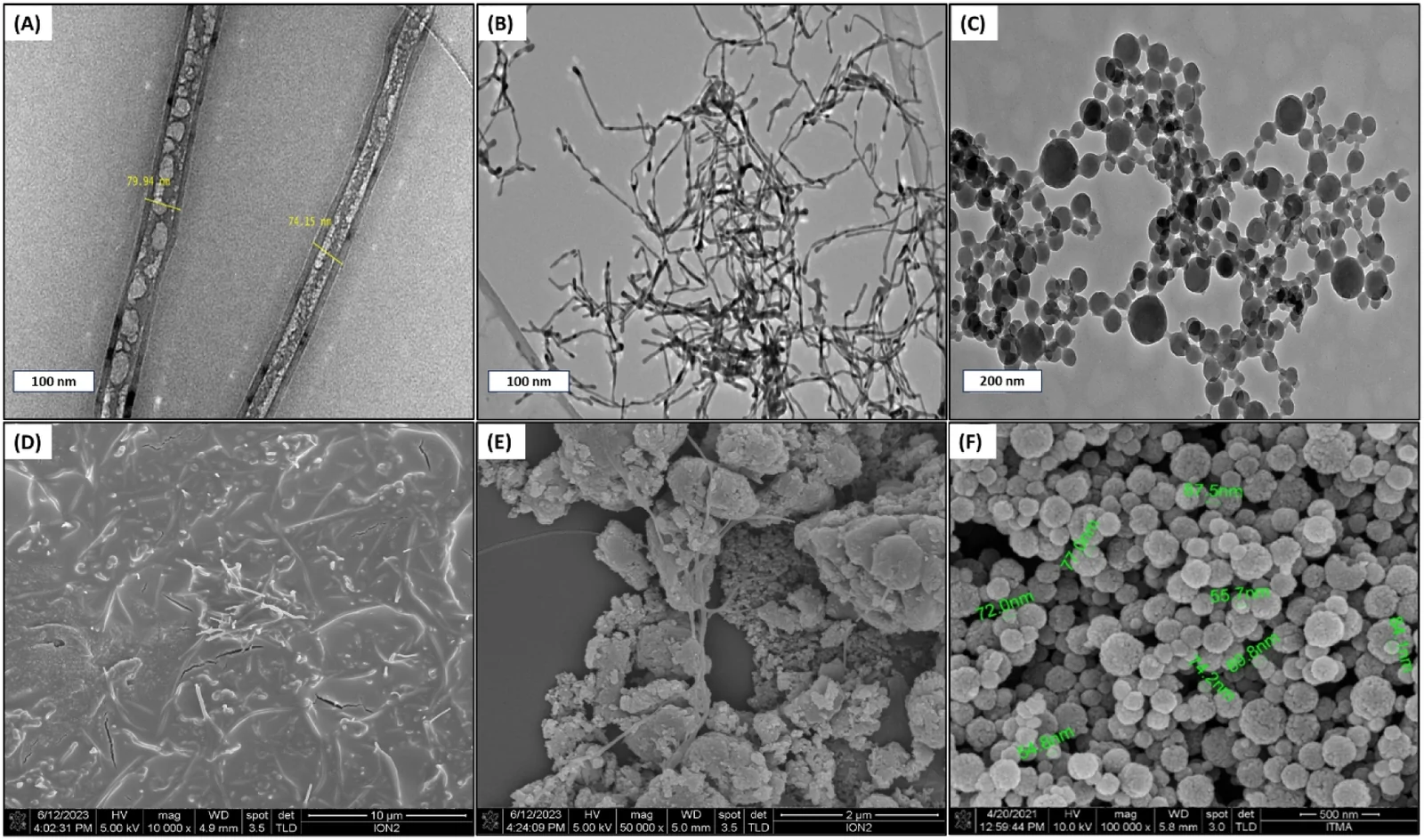
HRTEM images of (A) CNFs, (B) AuNWs, and (C) MIPs. FESEM images of (D) CNFs, (E) AuNWs, and (F) MIPs.

We investigated the structure and surface morphology of the modified electrodes (CNFs/AuNWs/MIPs/SPCE) using FESEM. This analysis examined changes in electrode surface morphology before and after drop-casting with bare SPCE, CNFs/SPCE, CNFs/AuNWs, CNFs/AuNWs/MIPs, and CNFs/AuNWs/NIPs, as illustrated in Figure 3.

**Figure 3:**
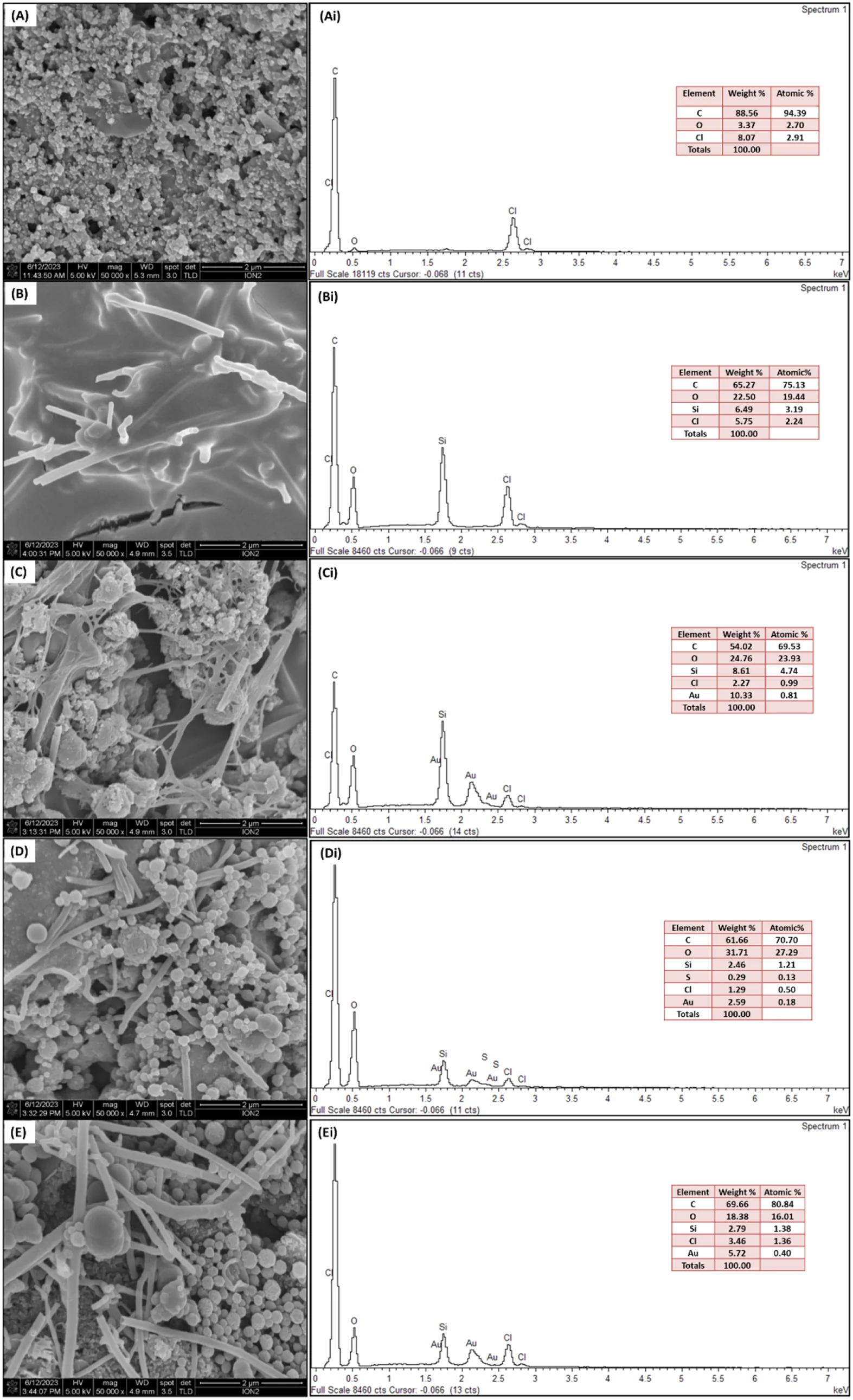
FESEM images of (A) bare SPCE, (B) CNFs/SPCE, (C) CNFs/AuNWs/SPCE, (D) CNFs/AuNWs/MIPs/SPCE, (E) CNFs/AuNWs/NIPs/SPCE, and EDX images of (Ai) bare SPCE, (Bi) CNFs/SPCE, (Ci) CNFs/AuNWs/SPCE, (Di) CNFs/AuNWs/MIPs/SPCE, and (Ei) CNFs/AuNWs/NIPs/SPCE at a magnification of 50 k.

Figure 3A presents the morphology of the bare SPCE, which exhibits the characteristic granular and relatively rough surface of the unmodified electrode. Following modification with CNFs, a distinct fibrous morphology was observed in Figure 3B, indicating the deposition of CNFs on the SPCE surface. The CNFs were distributed irregularly over the electrode surface, forming a conductive fibrous network. Further modification with AuNWs resulted in the appearance of elongated, wavy nanowire-like structures on the CNF-modified electrode (Figure 3C). The AuNWs exhibit several bends and kinks along their length and are distributed among the CNF network. This combination of fibrous CNFs and AuNWs produces a rough, three-dimensional surface that increases the electrochemically active area and facilitates electron transport.

The morphology of the CNFs/AuNWs/MIPs-modified electrode (Figure 3D) indicates numerous spherical structures distributed over the underlying fibrous network. These spherical features are attributed to MIP layer formation on the CNF/AuNW-modified surface. A relatively heterogeneous distribution of the polymer structures is evident. This heterogeneous morphology is expected because the electrode modification used a drop-casting approach rather than electrodeposition.^26–28^ During drop casting, the deposited materials can undergo localized aggregation and nonuniform distribution upon solvent evaporation, particularly for nanostructured materials such as CNFs and AuNWs. Similar spherical polymeric features were observed on the nonimprinted polymer-modified electrode (Figure 3E), supporting polymer layer formation on the CNF/AuNW-modified surface.

Although the modified surface does not exhibit a completely homogeneous distribution, distinct morphological changes are evident after each modification step. Together with elemental composition from EDS, FTIR, and electrochemical characterization, these provide complementary evidence supporting successful fabrication of the CNFs/AuNWs/MIPs-modified SPCE. The presence of Au in the EDS spectra of the AuNW-containing electrodes further supports the incorporation of AuNWs into the composite electrode. The heterogeneous nanostructured morphology from the drop-casting process may also create a rough, high-surface-area interface that benefits electrochemical sensing.

The EDX spectra for all modification steps are illustrated in Figure 3Ai–Ei. Figure 3Ai illustrates that carbon (C) is 88.56% and oxygen (O) is 3.37% on the bare SPCE. In Figure 3Bi, the percentage of oxygen (O) increased to 22.50%, confirming the successful introduction of functionalized CNFs onto the working surface of the bare electrode. Additionally, in Figure 3Ci, the gold (Au) element is detected at 10.33% in the EDX spectrum, indicating the successful fabrication of AuNWs onto the CNFs/SPCE-modified electrodes. In Figure 3Di, the increasing percentages of carbon (C) and oxygen (O) at 61.66% and 31.71%, respectively, indicate the distribution of MIPs on CNFs/AuNWs/SPCE. Furthermore, the presence of the sulfur (S) peak at 0.29% in the CNFs/AuNWs/MIPs/SPCE’s EDX spectrum, along with C, O, and Au, verified the presence of the PenG template inside the MIPs’ chemical composition. Any sodium (Na) peak that is not firmly bound to the surface may be removed during cleaning of unbound materials, thereby contributing to its apparent absence in EDX analysis. Figure 3Ei also confirms the chemical distribution of NIPs in CNFs/AuNWs/NIPs/SPCE, with carbon (C) at 69.66% and oxygen (O) at 18.38%. The absence of sulfur (S) and sodium (Na) peaks confirms the presence of the PenG template in the NIPs’ chemical composition. In summary, the presence of C, O, Au, and S elements indicates successful fabrication of CNFs/AuNWs/MIPs on the working electrode, enabling sensitive and selective targeting of the analyte PenG in the electrochemical system. A small Cl peak was observed in the EDX spectra for all modification steps, which originates from the binder during its preparation.^20,21^ Additionally, it may be due to residual chloride species from electrode fabrication or the Ag/AgCl reference electrode, as is frequently reported for commercial SPCE.

### 3.2 Analytical Response of Modified SPCE

The electrochemical sensor fabrication process was monitored using cyclic voltammetry (CV), differential pulse voltammetry (DPV), and electrochemical impedance spectroscopy (EIS) techniques. Figure 4 displays a CV plot of [Fe­(CN)_6_]^3–^/^4–^ (5 mM) recorded at 100 mV/s during the modification process. As indicated in Figure 4A, the bare SPCE (red) exhibited a clear pair of well-defined redox peaks. Compared with the unmodified electrode, the CNFs/SPCE-modified electrode (green) exhibited a markedly higher current response. This indicates that the fibrous structure of CNFs enhances electron transfer at the electrode interface, primarily through increased surface area. A self-assembled monolayer (SAM) of DTDPA was further introduced on the surface of CNFs/SPCE to effectively link the AuNWs to the CNF surface via covalent bonding attachment. Ultimately, the CNFs/AuNWs/SPCE (blue) indicates the highest current response among all electrodes tested, surpassing both the unmodified and other modified electrodes, suggesting enhanced electron-transfer kinetics due to the excellent conductivity of AuNWs.

**Figure 4:**
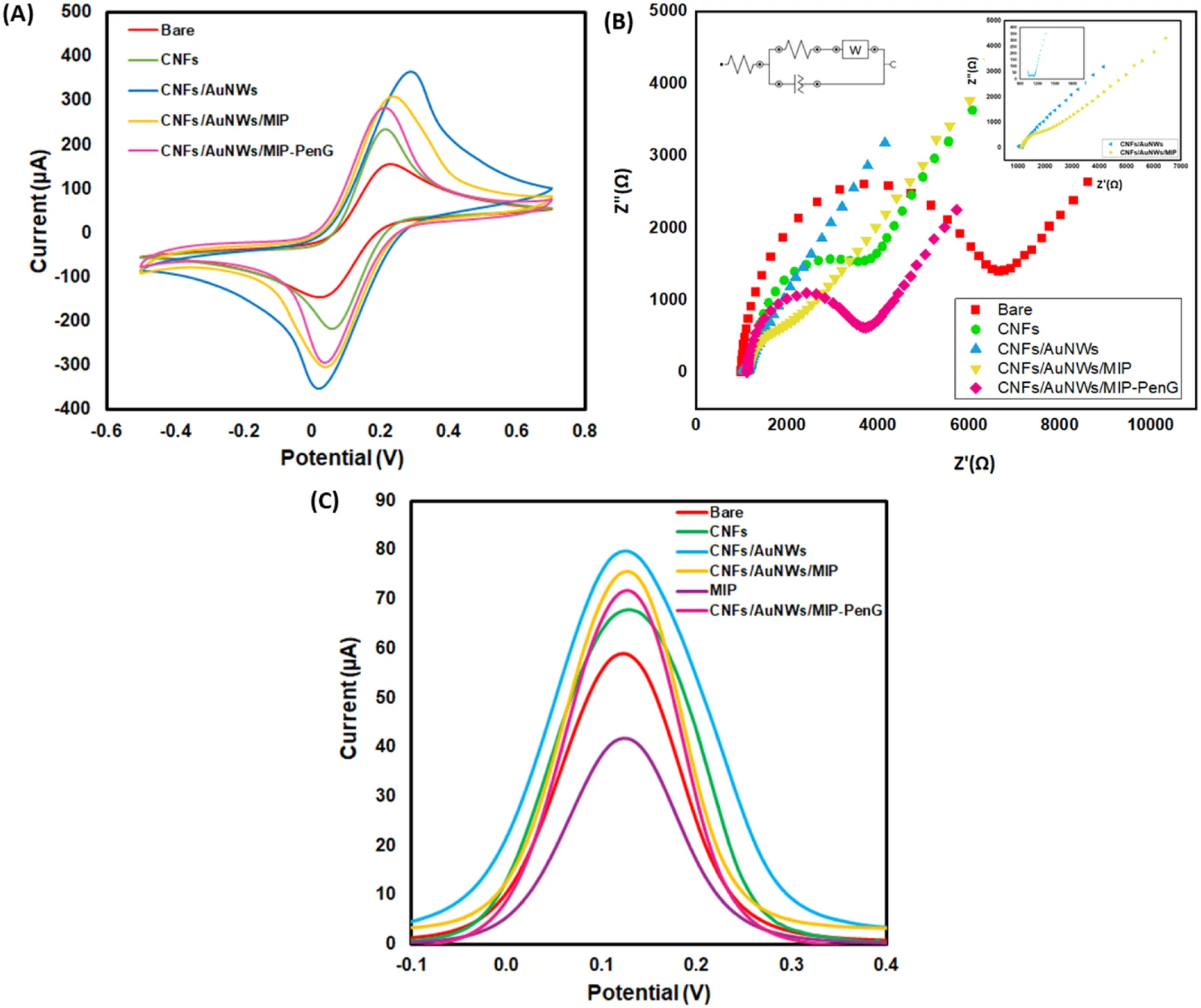
(A) CV, (B) EIS, and (C) DPV characterization of the stepwise modified electrode in 5 mM [Fe­(CN)_6_]^3^–/^4–^ containing 0.1 M KCl and 0.001 μM PenG.

In addition, the prepared nanocomposite-modified electrode exhibited the highest peak current due to the increased number of active sites for the detection of PenG. In contrast, CNFs/AuNWs/MIPs/SPCE (yellow) exhibited a drop in current response. The peak current gradually decreased when MIP was immobilized onto CNFs/AuNWs/SPCE, due to the MIP’s insulating nature, which hinders electron transfer. Furthermore, the peak current gradually decreased after immobilization of the target analyte, PenG. The adsorption of the target analyte, PenG, resulted in the imprinted cavities selectively adsorbing PenG molecules, thereby blocking electron-transfer channels and causing a subsequent decrease in peak current.

Furthermore, EIS provides insights into changes in electrode surface impedance during the modification process. This technique can effectively characterize the interfacial properties of the modified electrode.^29^ The interface is typically represented by an equivalent circuit comprising the electrolyte’s ohmic resistance (R_s_), the Warburg impedance (Z_w_), the electron-transfer resistance (R_ct_), and a constant phase element (CPE). In the electrochemical circuit model (ECM), R_s_ presents the electrolyte solution resistance, and R_ct_ represents the resistance of the electrochemical reaction at the electrolyte-electrode interface. In addition, Z_w_ is defined as the impedance of ion diffusion to the electrode. CPE is expressed as the capacity of the electrochemical reaction that occurs at the electrode–electrolyte interface and the current distribution on the electrode.^30^

Figure 4B illustrates the Nyquist plot for the bare SPCE, CNFs/SPCE, CNFs/AuNWs/SPCE, CNFs/AuNWs/MIP, and CNFs/AuNWs/MIP-PenG electrodes in 5 mM of [Fe­(CN)_6_]^3–^/^4–^ and 0.1 M KCl. The Randles circuit was introduced to fit the impedimetric results demonstrated in the inset of Figure 4B. The EIS response features a semicircular region and a linear region. The semicircular portion observed at higher frequencies corresponds to the electron-transfer-limited process, with its diameter representing R_ct_. In contrast, the linear segment at lower frequencies reflects the diffusion process. The bare SPCE highlights a larger semicircle (red), indicating higher resistivity and weak electrical conductivity. The R_ct_ value for the bare SPCE was 5190 Ω. After the fabrication of CNFs (green) onto the electrode surface of bare SPCE (CNFs/SPCE), the R_ct_ value decreased to 3520 Ω. Furthermore, the resistivity of CNFs/AuNWs/SPCE (blue) decreases drastically, providing an R_ct_ of 126 Ω. Based on the observation, the Nyquist plot shows a linear segment with a very small semicircle (inset), demonstrating the excellent electron-transfer properties of AuNWs. These findings are consistent with expectations, as the nanostructures of the AuNWs increase the electrode’s surface area and enhance its electrocatalytic activity. On the other hand, the increment of the R_ct_ (524 Ω) of the CNFs/AuNWs/MIPs-modified electrode (yellow), as indicated by an increase in semicircle, may be attributed to the insulating capabilities of MIPs.

Furthermore, the immobilization of PenG molecules onto the MIPs structure enhances the resistivity of the CNFs/AuNWs/MIPs/SPCE, with R_ct_ of 1036 Ω, suggesting successful recognition of the polymer matrices and their target analyte. In addition, the DPV analysis in Figure 4C aligns with the CV and EIS results at each step of the modification process. The current response and DPV behavior of all modified electrodes are similar under the same conditions. The performance of the electrochemical sensor depends on the effective surface area of the modified electrode, which can be calculated using the Randles-Sevcik equation.^31^

$$I_{p}=\left(2.69\times 10^{5}\right)n^{3/2}AC\sqrt{D\nu }$$

where I_p_ represents anodic peak current, n is the number of electron transfer in the redox event (n = 1), A is the electroactive surface area, D is the [Fe­(CN)_6_]^3–^/^4–^ diffusion coefficient (7.6 × 10^–6^ cm^2^ s^–1^), C is the concentration of [Fe­(CN)_6_]^3–^/^4–^ solution, and ν refers to the square root of scan rate.

CV technique was utilized to measure the unmodified and modified electrodes at different scan rates (10 mVs^–1^–100 mV^s–1^) with 5.0 mM [Fe­(CN)_6_]^3–^/^4–^ containing 0.1 M KCl solution as noted in Figure 5. The effective surface area of the bare SPCE in Figure 5A was calculated to be 0.0472 cm^2^, whereas that of the modified SPCE increased to 0.1333 cm^2^. The results indicate that applying CNFs/AuNWs nanomaterials to the electrode surface increased the effective surface area by approximately 2.82-fold relative to the unmodified SPCE, thereby improving electrochemical sensor signal detection. This surface-area enhancement is particularly advantageous for the sensitivity and efficacy of molecularly imprinted polymer electrochemical sensors. As illustrated in Figure 5B, the redox peak current observed for the modified SPCE exhibited a linear increase with the square root of the scan rate $\left(\sqrt{\nu }\right)$ within the range of 10 to 100 mV/s. This behavior strongly suggests that a standard diffusion-controlled process governs the electrochemical reaction involving ferricyanide. This correlation underscores the importance of electrode modification in enhancing the sensor’s electrochemical responsiveness.

**Figure 5:**
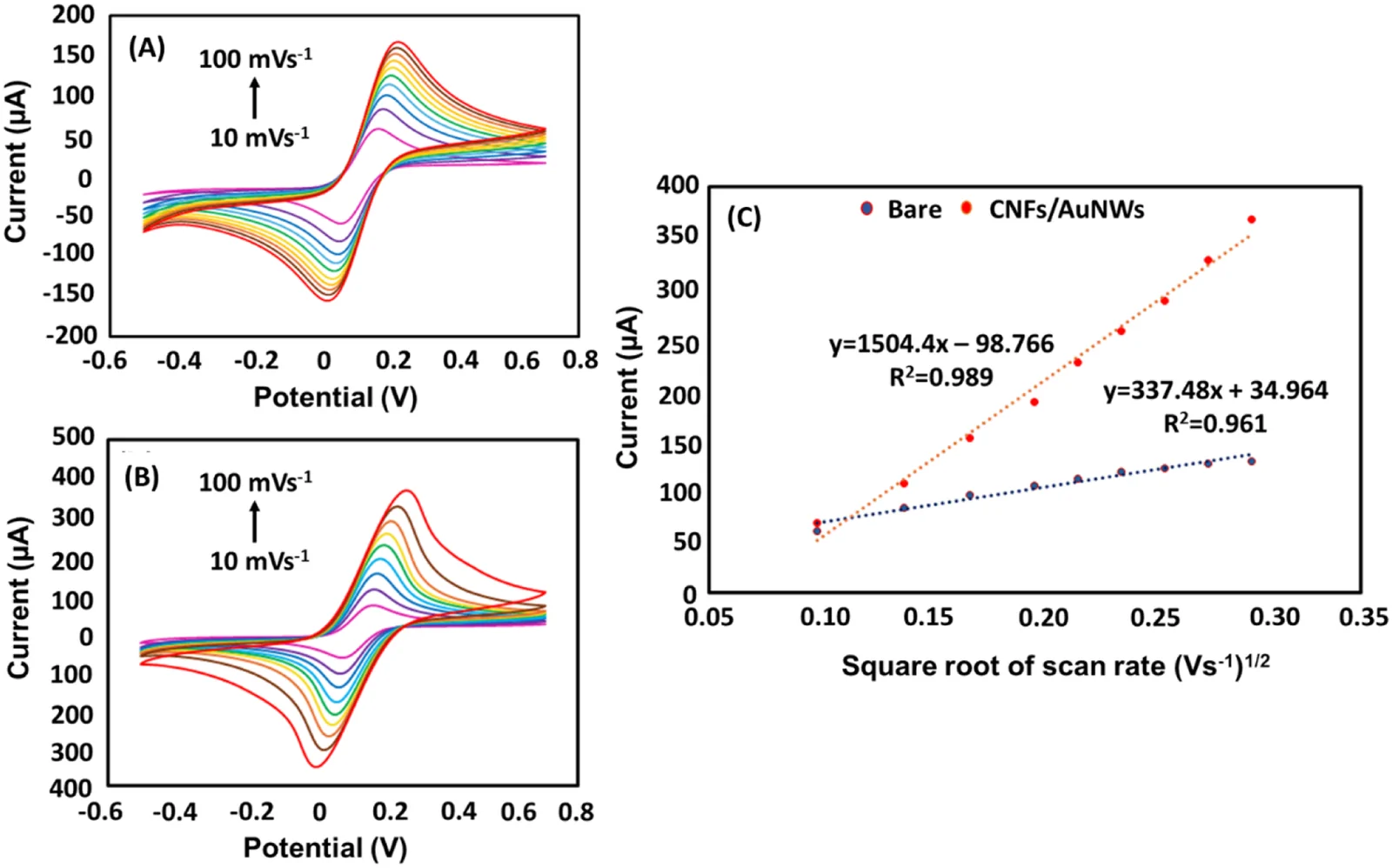
CV response of (A) bare SPCE, (B) CNFs/AuNWs-SPCE in 5.0 mM [Fe­(CN)_6_]^3–^/^4–^ in the presence of 0.1 M KCl solution over a wide range of scan rate 10 mV s^–1^ to 100 mV s^–1^, and (C) plot of peak current vs square roots of scan rate (*n* = 3).

### 3.3 Analytical Performance of PenG Biosensor

The DPV method was used to analyze the peak current of [Fe­(CN)_6_]^3–^/^4–^ after PenG adsorption on CNFs/AuNWs/MIPs/SPCE, thereby evaluating the detection performance of the developed sensor. The results are presented in Figure 6. Based on the observations, the detected current consistently decreased with increasing PenG concentration, as demonstrated in Figure 6A. This decrease is attributed to PenG occupying the original imprinted cavities, thereby hindering electron transfer and reducing the current. The calibration curve was constructed to assess the linearity of the [Fe­(CN)_6_]^3–^/^4–^ peak current using different PenG concentrations. As illustrated in Figure 6B, the curve is highly linear, with a linear regression coefficient of 0.9892. The limit of detection (LOD) of the developed CNFs/AuNWs/MIPs-PenG/SPCE electrochemical sensor was determined to be 1.0 nM.

**Figure 6:**
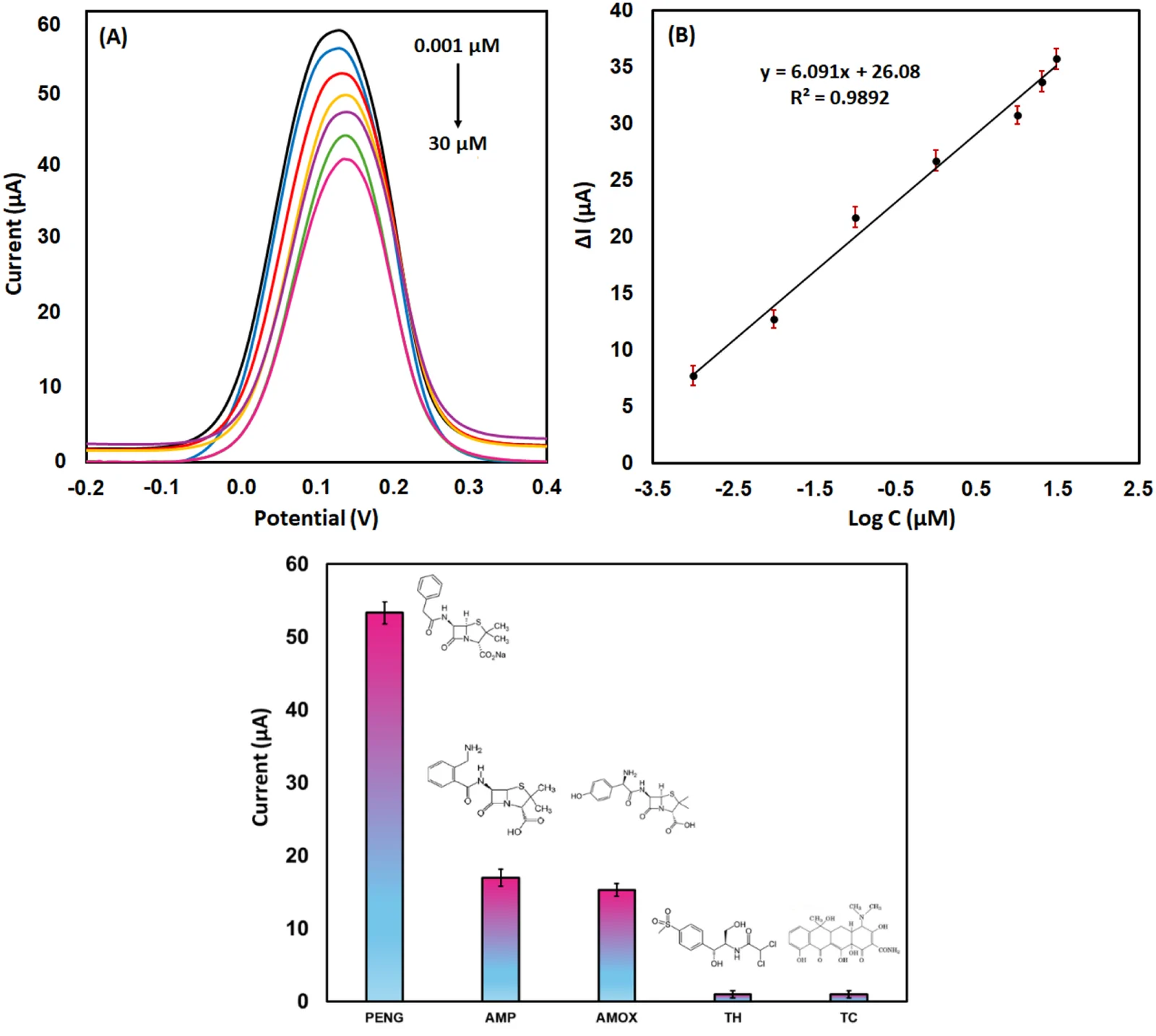
(A) DPV voltammograms of CNFs/AuNWs/MIPs/SPCE in the different concentrations of PenG. (B) A linear plot of Δpeak current against logarithmic value of different concentrations (n = 3). (C) Selectivity of developed sensor CNFs/AuNWs/MIPs/SPCE.

The selectivity of the CNFs/AuNWs/MIPs/SPCE toward PenG was investigated by introducing structurally similar penicillin analogues, such as ampicillin (AMP) and amoxicillin (AMOX). Additionally, some nonpenicillin antibiotics, including thiamphenicol (TH) and tetracycline (TC), were also assessed. Figure 6C indicates the selectivity results, indicating that the developed electrochemical sensor exhibits high selectivity for its target molecule, PenG. In contrast, low selectivity was observed for other β-lactam antibiotics, including AMP and AMOX, as well as nonpenicillin antibiotics, such as TH and TC. According to the results, the highest peak current (53.3 μA) was observed with PenG, followed by AMP (17.0 μA), AMOX (15.3 μA), TH (1.0 μA), and TC (1.0 μA). This finding revealed that this developed molecularly imprinted polymer-based electrochemical sensor can recognize its target molecules and discriminate between penicillin and nonpenicillin antibiotics.

Meanwhile, the repeatability of the fabricated sensor was also evaluated through five repetitive measurements of a single CNFs/AuNWs/MIPs/SPCE electrode in 0.1 μM PenG, as indicated in Figure 7A. The result demonstrates that the fabricated electrode maintains 80% of the recovery rate by the fourth cycle. Additionally, the stability of the CNFs/AuNWs/MIPs was studied by recording the current each week, as illustrated in Figure 7B. The modified electrode was stored at room temperature. From the results obtained, the current responses maintained 90% of the recovery by week four (28 days). Furthermore, the sensor reproducibility indicated a relative standard deviation (RSD) of 1.14%, suggesting that the fabricated sensors are reproducible.

**Figure 7:**
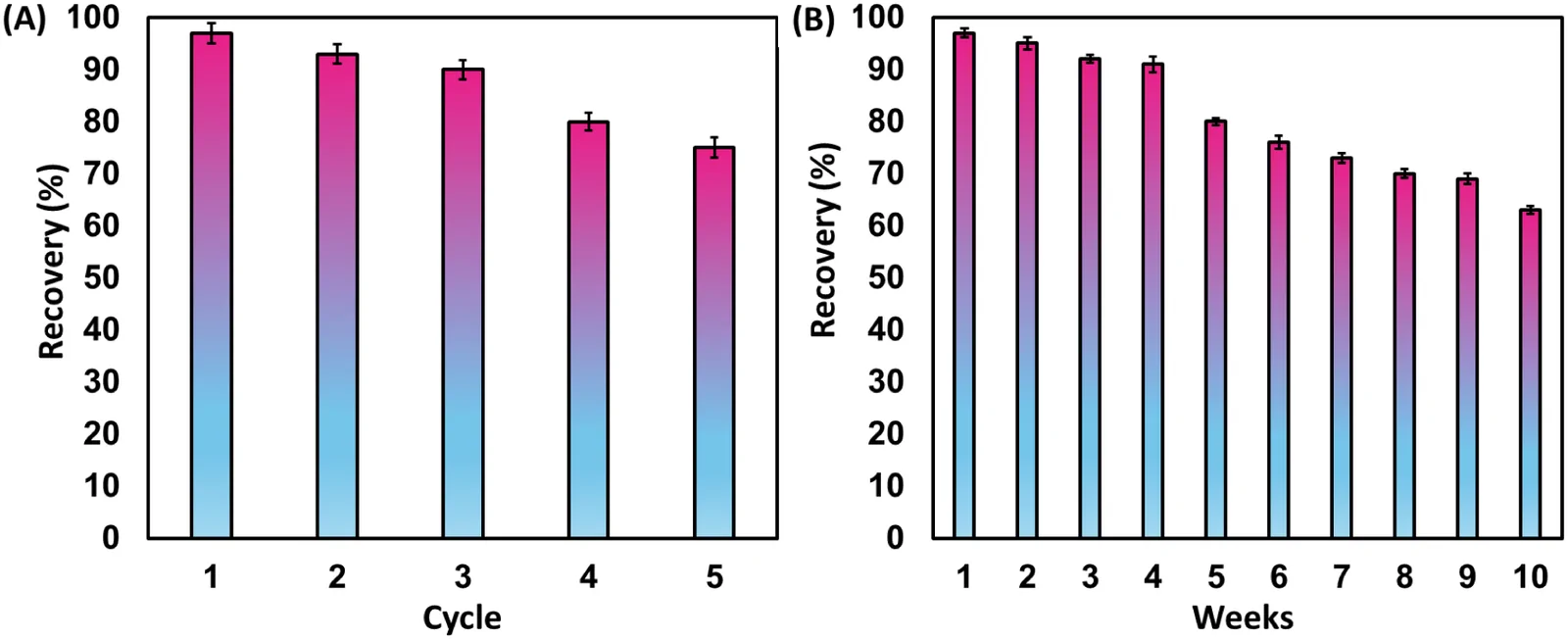
(A) Repeatability and (B) stability of the CNFs/AuNWs/MIPs/SPCE.

### 3.4 Real Sample Applications

The practical application of CNFs/AuNWs/MIPs/SPCE for detecting PenG in nonpenicillin pharmaceutical formulations was successfully demonstrated through comprehensive testing of a common pharmaceutical formulation, sulfamethoxazole+trimethoprim (SMX-TMX). To validate the detection method, the SMX-TMX drug was intentionally spiked with varying concentrations of PenG, as outlined in Table 1. The results indicated that the CNFs/AuNWs/MIPs-PenG/SPCE exhibited recovery rates of 94%–95%. These recovery rates confirm the accuracy and robustness of the detection method, highlighting its practical applicability in real-world pharmaceutical industries.

**Table 1:** Recoveries of CNFs/AuNWs/MIPs-PenG/SPCE in Pharmaceutical Drug Products

Pharmaceutical Drug Product	Spiked (μM)	Found (μM)	Recovery (%)
	0.50	0.47 ± 0.02	94 ± 0.52
Sulfamethoxazole+trimethoprim (SMX-TMX)	1.00	0.96 ± 0.01	96 ± 1.00
	1.50	1.43 ± 0.02	95 ± 1.00

The analytical performance of the developed CNFs/AuNWs/MIPs/SPCE electrochemical sensor was compared with previously reported Penicillin G sensors, as summarized in Table 2. The literature reports relatively few MIP-based electrochemical sensors for Penicillin G detection. The proposed sensor exhibited a low detection limit of 1.0 nM and a broad linear range of 0.001–30 μM, demonstrating promising analytical performance. From a materials perspective, the combination of CNFs and AuNWs provides a conductive, high-surface-area network that enhances electron transfer. In contrast, the MIPs provide selective molecular recognition of Penicillin G.^32,33^ This synergistic combination of enhanced electrochemical transduction and molecular selectivity gives the proposed CNFs/AuNWs/MIPs/SPCE platform an important advantage over conventional MIP-based Penicillin G sensors.

**Table 2:** Comparison of the Developed Biosensors toward Penicillin G Detection

Developed Sensor	Detection Technique	Real Sample	Linear Range	LOD	Ref
CPE/MIP	SWV	Blood plasma	5.0 × 10^–8^–1.0 × 10^–6^ M	3.8 × 10^–8^ M	^34^
MICP	EIS	Milk	12.5–100 ppb	-	^35^
FeS_2_@C/MIECS	DPV	-	0.5 μM–1 mM	-	^36^
AuNWs/CNFs/MIPs	DPV	Pharmaceutical drug products	0.001 μM–30 μM	1.0 nM	This work

## 4 Conclusions

A novel NMIP-based electrochemical sensor for penicillin G detection was successfully developed using CNFs and AuNWs modified on a SPCE. The fabricated electrodes were electrochemically characterized using CV, EIS, and DPV, with ferri-ferrocyanide [Fe­(CN)_6_]^3–^/^4–^ employed as the redox probe. Furthermore, the developed sensors were morphologically characterized using FESEM, HRTEM, and EDX analyses. The findings demonstrated that the sensor effectively rebinds penicillin G over a concentration range of 0.001–30 μM, indicating the presence of specific binding sites. These electrochemical results demonstrate the high sensitivity of the CNFs/AuNWs/MIPs/SPCE sensor and highlight its potential as a promising platform for penicillin G detection.

## Data Availability

The data underlying this study are available in the published article.
